# The construction of the Split Sleep Questionnaire on sleep habits during the COVID-19 pandemic in the general population

**DOI:** 10.3325/cmj.2022.63.299

**Published:** 2022-06

**Authors:** Linda Lušić Kalcina, Ivana Pavlinac Dodig, Renata Pecotić, Sijana Demirović, Maja Valić, Zoran Đogaš

**Affiliations:** 1 Department of Neuroscience, University of Split School of Medicine, Split, Croatia; 2 Sleep Medicine Center, University Hospital Split and University of Split School of Medicine, Split, Croatia; 3 Department for Neuroscience, University of Mostar School of Medicine, Mostar, Bosnia and Herzegovina

## Abstract

**Aim:**

To construct a single-format questionnaire on sleep habits and mood before and during the COVID-19 pandemic in the general population.

**Methods:**

We constructed the Split Sleep Questionnaire (SSQ) after a literature search of sleep, mood, and lifestyle questionnaires, and after a group of sleep medicine experts proposed and assessed questionnaire items as relevant/irrelevant. The study was performed during 2021 in 326 respondents distributed equally in all age categories. Respondents filled out the SSQ, the Pittsburgh Sleep Quality Index (PSQI), and State Trait Anxiety Inventory (STAI), and kept a seven-day sleep diary.

**Results:**

Workday and work-free day bedtime during the COVID-19 pandemic assessed with SSQ were comparable to the sleep diary assessment (*P* = 0.632 and *P* = 0.203, respectively), as was the workday waketime (*P* = 0.139). Work-free day waketime was significantly later than assessed in sleep diary (8:19 ± 1:52 vs 7:45 ± 1:20; *P* < 0.001). No difference in sleep latency was found between the SSQ and PSQI (*P* = 0.066). Cronbach alpha for Sleep Habits section was 0.819, and 0.89 for Mood section. Test-retest reliability ranged from 0.45 (*P* = 0.036) for work-free day bedtime during the pandemic to 0.779 (*P* < 0.001) for sleep latency before the pandemic.

**Conclusion:**

The SSQ provides a valid, reliable, and efficient screening tool for the assessment of sleep habits and associated factors in the general population during the COVID-19 pandemic.

The COVID-19 pandemic, along with its multiple adverse effects on various aspects of mental health, has significantly affected sleep. Sleep habits alterations and newly developed sleep disturbances during the COVID-19 pandemic may influence the overall well-being and health (1). Since the beginning of the pandemic, several studies reported a delay in bedtimes and waketimes, and an associated shift in chronotype toward eveningness (2-5).

Even though actigraphy and sleep diaries provide a valid and reliable assessment of sleep habits (6,7), to achieve the highest reliability and validity, these methods require an assessment during seven consecutive days including weekends (8). Daily reporting may be perceived by the respondents as an additional burden (6,9), a limitation that may be overcome by the use of single-administration questionnaires (9,10). Since sleep disturbances recognized in the first pandemic outbreak remained stable during new waves of the COVID-19 pandemic (5), single-administration questionnaires may enable screening of large population groups and an extended assessment of sleep disturbances during the pandemic.

So far, validated sleep questionnaires have most often aimed at sleep disorders or symptoms associated with sleep disorders (9). Studies commonly report the Pittsburgh sleep Quality Index (PSQI) (11), which provides data on sleep duration, sleep disturbances, and sleep latency during the previous month. However, PSQI reflects mainly sleep quality on workdays (12), while not collecting information on sleep habits on weekends. The Sleep Timing Questionnaire (STQ) has been developed as an alternative to the sleep diary for the healthy adult population, showing good reliability and validity (10). Still, although sleep habits are associated with mood (13), social media use (14-16), learning time in students (17-19), sports or exercise (20), and symptoms of insomnia (21), the STQ does not assess variables such as mood and lifestyle habits.

Large studies objectively assessing sleep with wearable devices have recognized sleep timing and sleep duration to be modifiable risk factors for adverse mental health during the current pandemic (22). Young adults are especially at risk for increased mood disorder symptoms, higher levels of perceived stress, and more common alcohol use during the pandemic (23). Even though mood disorders are often reported in pandemic studies on sleep habits, mood itself has been less commonly measured and associated with sleep parameters (24). A review of the literature showed a transactional relationship between mood and emotion (25), indicating that mood is characterized by longer duration than emotion (26). Mood is often assessed with the Brief Mood Introspection Scale (27), the Profile of Mood States (28), or the Visual Analogue Mood Scale (29). A relevant aspect of mood measurement is a hierarchical structure with two broad dimensions in positive and negative affect, and multiple specific states (30). Commonly used mood assessment scales evaluate the basic negative mood of fear/anxiety, sadness/depression, and anger/hostility, as well as at least one positive mood. Therefore, it has been strongly recommended that mood researchers assess a broad range of both positive and negative emotions (30).

Linking mood changes and lifestyle habits during the pandemic has been relevant in order to recognize possible predictors of mood changes, especially due to a reported increase in depression (31). Since sleep is often intertwined with mood and lifestyle changes (31), we assumed that a single-format questionnaire comprehensively assessing these variables and sleep may be applicable and timely.

The aim of this study was to construct a single-format Split Sleep Questionnaire (SSQ) comprehensively assessing sleep habits, lifestyle habits, and mood changes, as well as to evaluate its reliability and validity in the general population. Sleep habits were validated by using standard instruments such as sleep diary, PSQI, and STAI questionnaires as the measures of construct validity. Additionally, we aimed to assess the psychometric properties of the Mood section and to explore the effects of the COVID-19 pandemic on sleep habits and mood alterations in the general population of Croatia.

## Respondents and methods

### Respondents

The study was performed in Croatia and Bosnia and Herzegovina during January and February 2021. An online form of the questionnaire was distributed to 326 respondents. A total of 269 respondents were citizens of Bosnia and Herzegovina, and 57 respondents were Croatian citizens. In both countries, respondents reported to live in both rural and urban areas. The study protocol was approved by the Biomedical Research Ethics Committee at the University of Split and University of Mostar School of Medicine. At the beginning of the survey, the participants were informed about the research aim and explained that their anonymity was protected. The sleep diary data anonymity was ensured by the use of personalized codes.

### Methods

Convenience sampling was used. A Google Form was distributed to the respondents via email or social media platforms. Respondents of different age groups were contacted in order to achieve sample heterogeneity. A web-based questionnaire enabled a fast distribution during a time when extended epidemiological restrictions and recommendations were in place. In order to validate the questionnaire, respondents were asked to fill out the PSQI (32) and STAI questionnaires (33), and to keep a sleep diary for seven days during January and February 2021. A standard form of seven-day diary was used (34).

### Instrument

*Item selection*. In order to identify factors associated with sleep quality during the pandemic and enable the item selection, we reviewed the literature in the area of behavioral sleep medicine. The literature search aimed at a comprehensive overview of sleep, mood, and lifestyle questionnaires. Sleep medicine experts were invited to a group discussion, where they proposed questionnaire items and assessed them as relevant/irrelevant. Group members were a European Sleep Research Society (ESRS) board-certificated somnologist, four medical doctors with clinical experience in a sleep medicine center, a psychologist experienced in sleep medicine, and an ESRS board-certified sleep technician. Items were included in the questionnaire if all members agreed upon them. Questions were excluded from the final form and new questions included if all members of the group agreed. Face validity and content validity were assessed in a group discussion.

Following the construction of the final version of the questionnaire, we conducted a study to assess its psychometric properties. Factorial validity was assessed in the areas with more than one dimension forming the investigated construct. Reproducibility was assessed for all sections with a test-retest intraclass correlation coefficient, calculated based on the questionnaire responses of 22 respondents filling out the same version of the questionnaire after 30 days. When possible, the Cronbach’s alpha was calculated in order to test the internal consistency. It was calculated only in the questionnaire sections aimed at assessing the consistency of individual responses to each item with the remaining items.

*Structure of the SSQ*. The final version of the SSQ consisted of sections assessing Personal Data (17 questions), Lifestyle Habits (31 questions), Sleep Habits (26 questions), and Mood (16 questions) before and during the COVID-19 pandemic (Supplementary Material(Supplementary Material)).

The first section collected personal data (year of birth, sex, weight, height, residence, university and school in the case of students, employment status, pandemic restrictions adherence etc). The Lifestyle Habits section inquired about cigarette smoking, coffee consumption, alcoholic beverages consumption, and exercise habits before and during the pandemic. It also included questions about the time spent learning (for students), watching TV, working on the computer, and using social networks and mobile phones before and during the pandemic. The Sleep Habits section consisted of questions about sleep timing before and during the pandemic, on workdays and work-free days. The respondents were also asked about sleep latency before and during the pandemic, sleeping habits during the day, and symptoms of sleep disturbances or insomnia. The Mood section inquired about the frequency of irritability, fear, rest, sadness, discouragement, satisfaction, anxiety, anger, and calm before and during the pandemic assessed on a Likert scale: 1 – almost never, 2 – sometimes, 3 – often, and 4 – almost always.

### Statistical analysis

Descriptive data are reported for the full sample with basic demographic information. Continuous variables are reported as mean (standard deviation) or median (interquartile range), whereas categorical variables are reported as frequencies (percentages). Positive or negative asymmetric data assessed with a significant Shapiro Wilks test underwent a parametric analysis due to a large sample size and the comparison of the variances of the compared variables. Q-Q plots and skewness or kurtosis of asymmetrically distributed data were inspected. ANOVA for repeated measures with post-hoc Bonferroni tests was used to test the differences between the SSQ, PSQI, and sleep diary assessment in the same respondents. A *t* test for paired samples was used to assess the changes between before and during the pandemic in the assessment with different tools in a single respondent. A test-retest correlation was reported for the items of Sleep Habits and Mood section. A factor analysis was performed for Sleep Habits and Mood Section, and Cronbach alpha coefficient was calculated. Structure matrix of the sections before and during the pandemic was reported following the Varimax rotation with Kaiser normalization. The criterion for factor selection was eigen >1. Data analysis was performed with SPSS, version 14 (SPSS, Chicago, IL, USA).

## Results

The analysis included 326 respondents (Table 1). The largest proportion of respondents was in the age group of 50 to 75 years (31.3%).

**Table 1:** Respondents’ demographic characteristics*

	Total sample
Sex	
Male	189 (58)
Female	137 (42)
Weight (kg)	77.8 ± 15.9
Height (cm)	175.4 ± 8.5
BMI (kg/m^2^)	25.2 ± 4.3
Age	49.8 ± 22.2
Age category	
<25 years	88 (27)
25 to 50 years	85 (26.1)
50 to 75 years	102 (31.3)
>75 years	51 (15.6)

*Categorical variables are presented as frequency (percentage). Continuous variables are presented as mean ± standard deviation. Age is presented as median (IQR).

### Factor analysis

**Figure 1:**
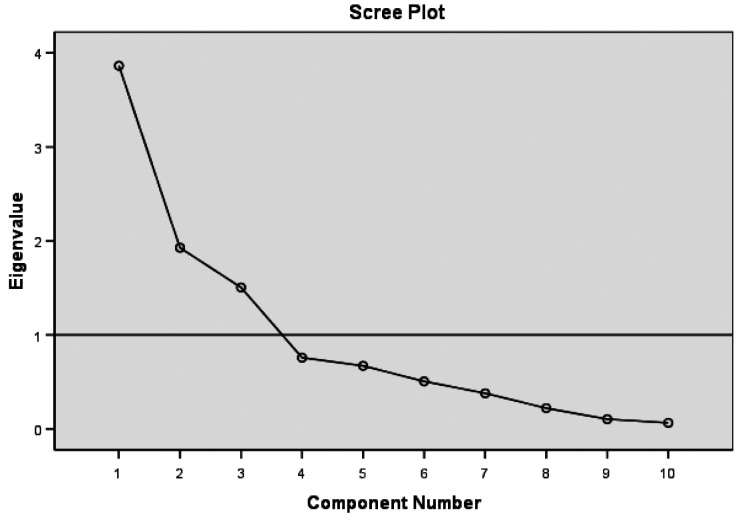
Scree plot of the Sleep Habits section.

The factor analysis of the Sleep Habits section (Table 2), revealed three factors explaining 72.96% of variance and having initial eigenvalues above 1 (Figure 1): bedtime (27.4% of variance), waketime (26.1% of variance), and sleep latency (19.4% of variance). Structure matrix (Table 3) revealed underlying variables of each factor.

**Table 2:** Factor analysis of the Split Sleep Questionnaire Sleep Habits section

Total variance explained								
Component	Initial Eigenvalues			Extraction SS loadings		Rotation SS loadings		
	Total	% of variance	Cumulative %	Total	% of variance	Total	% of variance	Cumulative %
Bedtime	3.86	38.63	38.63	3.86	38.63	2.74	27.43	27.43
Waketime	1.93	19.28	57.91	1.93	19.28	2.61	26.12	53.54
Sleep latency	1.50	15.05	72.96	1.50	15.05	1.94	19.41	72.96

**Table 3:** Structure matrix of the Split Sleep Questionnaire Sleep Habits section (rotation method: Varimax with Kaiser normalization)

	Bedtime	Waketime	Sleep latency
**Weekday bedtime before the COVID-19 pandemic**	0.806		
**Weekday bedtime during the COVID-19 pandemic**	0.837		
**Weekend bedtime before the COVID-19 pandemic**	0.768		
**Weekend bedtime during the COVID-19 pandemic**	0.699		
**Weekday waketime before the COVID-19 pandemic**		0.729	
**Weekday waketime during the COVID-19 pandemic**		0.822	
**Weekend waketime before the COVID-19 pandemic**		0.815	
**Weekend waketime during the COVID-19 pandemic**		0.795	
**Sleep latency before the COVID-19 pandemic**			0.969
**Sleep latency during the COVID-19 pandemic**			0.969

**Figure 2:**
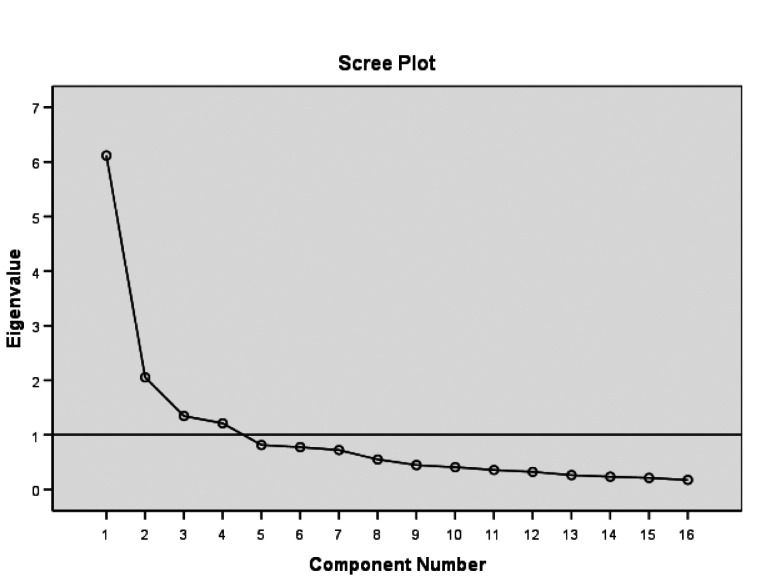
Scree plot of the Mood section.

PCA of the Mood section revealed four underlying factors with initial eigenvalues above 1 (Table 4 and Table 5), confirmed with a scree plot (Figure 2): sadness, discouragement, and fear before and during the pandemic (20.2% of variance); calm, rest, and satisfaction before and during the pandemic (20.04% of variance); anger and anxiety during the pandemic factor (15.1% of variance); and anger and anxiety before the pandemic (11.7% of variance). They together explained 67% of variance.

**Table 4:** Factor analysis of the Split Sleep Questionnaire Mood section before and during the pandemic

Factor	Initial Eigenvalues			Extraction SS loadings		Rotation SS loadings		
	Total	% of variance	Cumulative %	Total	% of variance	Total	% of variance	Cumulative %
Sadness, discouragement, and fear before and during pandemic	6.12	38.23	38.23	6.12	38.23	3.23	20.21	20.21
Calm, rest, and satisfaction before and during pandemic	2.05	12.84	51.07	2.05	12.84	3.21	20.04	40.25
Anger and anxiety during pandemic	1.35	8.41	59.48	1.35	8.41	2.42	15.12	55.37
Anger and anxiety before pandemic	1.21	7.57	67.05	1.21	7.57	1.87	11.68	67.05

**Table 5:** Structure matrix of the Split Sleep Questionnaire Mood section before and during the pandemic (rotation method: Varimax with Kaiser normalization)

	Sadness, discouragement, and fear before and during pandemic	Calm, rest, and satisfaction before and during pandemic	Anger and anxiety during pandemic	Anger and anxiety before pandemic
Afraid before the COVID-19 pandemic	0.749			
Discouraged before the COVID-19 pandemic	0.754			
Sad before the COVID-19 pandemic	0.675			
Afraid during the COVID-19 pandemic	0.547			
Discouraged during the COVID-19 pandemic	0.688			
Sad during the COVID-19 pandemic	0.701			
Calm before the COVID-19 pandemic		0.803		
Rested before the COVID-19 pandemic		0.791		
Satisfied before the COVID-19 pandemic		0.658		
Calm during the COVID-19 pandemic		0.639		
Rested during the COVID-19 pandemic		0.691		
Satisfied during the COVID-19 pandemic		0.653		
Anxious during the COVID-19 pandemic			0.740	
Angry during the COVID-19 pandemic			0.714	
Anxious before the COVID-19 pandemic				0.740
Angry before the COVID-19 pandemic				0.817

### Test-retest reliability and internal consistency

In the Sleep Habits section, we found a significant test-retest correlation of workday and work-free day bedtime and waketime assessment, as well as sleep latency assessment both before and during the COVID-19 pandemic (Table 6). Similarly, in the Mood section, a significant test-retest correlation of mood assessment time points was found before and during the pandemic (Table 7) in all variables except angriness before the pandemic (r = 0.253; *P* = 0.257). Cronbach alpha values were calculated for Sleep Habits section including all bedtime and waketime questions, with a value of 0.819. Additionally, Cronbach alpha value was reported for Mood section after recoding the variables that were coded in the opposite direction, achieving a value of 0.89. Internal consistency for Mood section was also assessed separately for mood before the pandemic and separately during the pandemic, being 0.79 and 0.85, respectively. Item-total statistics for Sleep Habits section and Mood section are reported in Supplementary Table 1(Supplementary Table 1) and Supplementary Table 2(Supplementary Table 2), respectively.

**Table 6:** Test-retest reliability of the Split Sleep Questionnaire Sleep Habits section (N = 326)

		Test-retest correlation	P
Before the COVID-19 pandemic	Workday bedtime	0.64	0.001
	Work-free day bedtime	0.721	<0.001
	Workday waketime	0.57	0.007
	Work-free day waketime	0.757	<0.001
	Sleep latency	0.779	<0.001
During the COVID-19 pandemic	Workday bedtime	0.553	0.008
	Work-free day bedtime	0.45	0.036
	Workday waketime	0.559	0.007
	Work-free day waketime	0.659	0.001
	Sleep latency	0.497	0.019

**Table 7:** Test-retest reliability of the Split Sleep Questionnaire Mood section (N = 326)

		Test-retest correlation	P
Before the COVID-19 pandemic	Calm	0.821	<0.001
	Rested	0.559	0.007
	Satisfied	0.801	<0.001
	Anxious	0.473	0.026
	Angry	0.253	0.257
	Afraid	0.683	<0.001
	Discouraged	0.73	<0.001
	Sad	0.558	0.007
During the COVID-19 pandemic	Calm	0.674	0.001
	Rested	0.72	<0.001
	Satisfied	0.556	0.007
	Anxious	0.783	<0.001
	Angry	0.573	0.005
	Afraid	0.527	0.012
	Discouraged	0.828	<0.001
	Sad	0.822	<0.001

### Sleep Habits section of the SSQ compared with sleep diary and PSQI results

Workday and work-free day bedtime during the COVID-19 pandemic assessed with the SSQ was comparable to sleep diary assessment (*P* = 0.632 and *P* = 0.203, respectively; Table 8). Workday waketime assessed with the SSQ was also comparable to sleep diary assessment (*P* = 0.139; Table 8), whereas work-free day waketime during the pandemic assessed with the SSQ was later than sleep diary assessment (8:19 ± 1:52 vs 7:45 ± 1:20; *P* < 0.001, respectively). Workday and work-free day bedtime assessments (*P* = 0.554 and *P* = 0.154, respectively), as well as workday waketime assessment (*P* = 0.053; Table 9), did not significantly differ between SSQ and PSQI.

**Table 8:** The Split Sleep Questionnaire (SSQ) compared with sleep diary sleep-wake time assessment*

		Assessment during the COVID-19 pandemic		
		Sleep diary	SSQ	P^†^
Bedtime	Workdays	22:58 ± 1:03	23:02 ± 2:10	0.632
	Work-free days	23:31 ± 1:25	23:25 ± 1:59	0.203
Waketime	Workdays	7:18 ± 1:04	7:25 ± 1:34	0.139
	Work-free days	7:45 ± 1:20	8:19 ± 1:52	<0.001

*Continuous variables are presented as mean ± standard deviation.

†t-test for paired samples.

**Table 9:** The Split Sleep Questionnaire (SSQ) compared with the Pittsburgh Sleep Quality Index (PSQI) sleep-wake time assessment*

		Assessment during the COVID-19 pandemic		
		PSQI	SSQ	P^†^
Bedtime	Workdays	23:05 ± 1:28	23:02 ± 2:08	0.554
	Work-free days		23:17 ± 2:58	0.154
Waketime	Workdays	7:35 ± 1:28	7:25 ± 1:34	0.053
	Work-free days		8:23 ± 1:51	<0.001

*Continuous variables are presented as mean ± standard deviation.

†t-test for paired samples.

**Figure 3:**
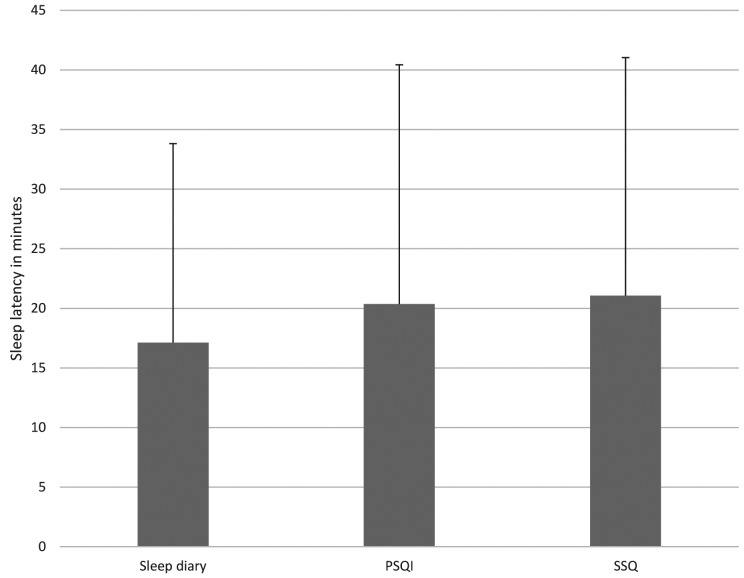
Sleep latency assessed with sleep diary and the Pittsburgh Sleep Quality Index (PSQI) and the Split Sleep Questionnaire (SSQ).

Sleep latency during the COVID-19 pandemic differed depending on the method of assessment (*P* < 0.001; Figure 3). The SSQ (21.06 ± 19.97 minutes; *P* < 0.001) and PSQI (20.36 ± 20.07 minutes; *P* < 0.001) showed higher sleep latency than sleep diary (17.13 ± 16.69 minutes), whereas no difference was found between the SSQ and PSQI assessments (*P* = 0.066).

Workday bedtime as well as both workday and work-free day waketime were significantly later during the pandemic than before the pandemic (Table 10). Bedtime on work-free days was the only sleep timing that remained stable before and during the pandemic (*P* = 0.351).

**Table 10:** Changes in the Split Sleep Questionnaire (SSQ) sleep-wake time assessment (before and during the COVID-19 pandemic)*

		SSQ		
		Assessment before the COVID-19 pandemic	Assessment during the COVID-19 pandemic	P†
Bedtime	Workdays	22:44 ± 1:25	23:02 ± 2:08	<0.001
	Work-free days	23:26 ± 1:58	23:17 ± 2:58	0.351
Waketime	Workdays	7:01 ± 1:32	7:25 ± 1:34	<0.001
	Work-free days	8:09 ± 1:40	8:23 ± 1:51	<0.001

*Continuous variables are presented as mean and standard deviation.

†t-test for paired samples.

A higher PSQI score was reported in respondents who in the SSQ reported problems with frequent awakenings (8.5 ± 2.52 vs 6.85 ± 2.11; *P* < 0.001), insomnia (9.02 ± 2.59 vs 7.11 ± 2.22; *P* < 0.001), or difficulties falling asleep during the pandemic (9.19 ± 2.56 vs 6.88 ± 2.05; *P* < 0.001). The correlation of the SSQ and sleep diary measures was significant for both bedtime and waketime on weekdays (r = 0.64, *P* < 0.001; r = 0.69, *P* < 0.001; respectively) and work-free days (r = 0.71, *P* < 0.001; r = 0.69, *P* < 0.001; respectively).

### Mood section – anxiety question comparison with the STAI results

Higher anxiety assessed with the STAI-S and STAI-T scales was reported in respondents reporting in the SSQ Mood section to be anxious almost always (STAI-*t* = 44.44 ± 16.9, STAI-S = 45.89 ± 16.79) and often (STAI-*t* = 44.14 ± 11.18, STAI-S = 43.17 ± 10.9) (*P* < 0.001). Lower sum scores on both scales were reported in respondents reporting to be anxious only sometimes (STAI-*t* = 35.21 ± 8.47, STAI-S = 34.94 ± 8.1) and almost never (STAI-*t* = 31.15 ± 8.85, STAI-S = 30.45 ± 7.55).

## Discussion

The novel SSQ yielded results comparable to sleep diary assessment. Additionally, the SSQ sleep habits assessment was comparable to the validated and widely used PSQI. More precisely, bedtime and waketime on workdays, as well as bedtime assessments on work-free days during the COVID-19 pandemic were comparable with sleep diary and PSQI reports. The SSQ enabled us to recognize a shift during the COVID-19 pandemic toward later bedtime and waketime on weekdays, and toward later waketime on work-free days. Waketime on work-free days during the COVID-19 pandemic was the only assessment reported as significantly later when compared with sleep diaries and the PSQI. A four-factor structure of the Mood section and a three-factor structure of the Sleep Habits section were revealed. Additionally, the current study showed a fair test-retest reliability in the Sleep Habits and Mood sections. Internal consistency was good for both the Sleep Habits section and the overall Mood section.

Workday and work-free day bedtime assessed with the SSQ were comparable with those assessed with sleep diary and PSQI questionnaire, which indicates good convergent validity of these items during the pandemic. However, the SSQ assessment of waketime on work-free days during the COVID-19 pandemic was later compared with sleep diary assessment. Indeed, sleep diary reflects respondents’ lifetime circumstances in a specific period, especially during a relatively short period of seven days. Contrarily, the SSQ enabled an overall subjective estimate of habitual bedtime and waketime, distinguishing the period before and during the COVID-19 pandemic. The pandemic environment influenced habitual bedtimes and waketimes, which often changed depending on the pandemic restrictions implemented in different countries (35,36). Presumably, later waketime reported on the SSQ compared with sleep diary reflects this pandemic environment. A comprehensive questionnaire such as the SSQ enabled us to recognize a shift toward later workday bedtime, as well as both workday and work-free day waketime during the pandemic, which has been previously recognized (35,36).

Our findings agree with previous studies that recognized such a shift in chronotype toward eveningness in adult population (2), small children (3), and high-school children (4). The SSQ enabled a valid assessment of sleep timing, which is relevant during the ongoing COVID-19 pandemic considering the association of sleep habits changes with public health outcomes (37-39). The use of the SSQ as a single-administration questionnaire in the general population may enable low-cost, easy administration, not requiring long-term commitment from respondents as is required for the use of sleep diaries or actigraphy devices.

COVID-19 is known to affect both sleep and mental health (36), and the current study adds to this knowledge by enabling a valid self-report assessment of some aspects of mood for the period before and during COVID-19 pandemic. The value of an added assessment of Mood for the time before and during the pandemic in the SSQ is relevant since 30% of middle-aged and older adults in Europe reported depressed mood, anxiety, or sleep problems during the pandemic (40). The SSQ enabled us to recognize four factors underlying mood before and during the pandemic, which may be differentiated as positive (calm, rest, and satisfaction before and during the pandemic) and negative (sadness, discouragement, and fear before and during the pandemic; anger and anxiety before the pandemic; anger and anxiety during the pandemic). Considering the absence of a structural consensus on the measurement of mood, we aimed to follow the recommendation that both positive and negative mood are assessed (30), and this was confirmed with factor analysis. We were able to assess the construct validity only for the question on anxiety, which was comparable to the results on the standardized STAI questionnaire. Additionally, internal consistency for Mood section reached 0.89 Cronbach alpha value, suggesting an overall good consistency.

Convergent validity, expressed as a positive association of the SSQ and sleep diary reports, was satisfactory for all sleep habits items, considering that only convergent validity below 0.50 is recommended to be avoided (41). Test-retest reliability was assessed during the pandemic, but reflected respondents' subjective assessments of the periods before and during pandemic. It varied from fair to excellent for different variables, based on the established norms for psychometric questionnaires (42). The only variable for which there was no significant association of the test-retest values was angriness assessment before the pandemic. One might assume that this assessment was more sensitive and influenced by additional factors, such as the current pandemic restriction measures or other unknown factors.

A decreased sleep quality as assessed with PSQI was reported. A sleep quality decrease was also detected by the SSQ, mainly in the form of frequent awakenings, newly developed insomnia, or difficulties falling asleep. Therefore, this section of the SSQ questionnaire also revealed good construct validity.

Among the limitations of the current study is the somewhat lower internal consistency for the Mood section during the pandemic (0.79), which should be interpreted with caution. A possible bias might also have resulted from the fact that no specific timeframe for duration of sleep before the pandemic was suggested for the respondents. Even though offering a detailed timeframe (the last week or the last month) may provide more specific information, the overall average self-reported assessment of sleep habits in both time periods (before and during the pandemic) is less affected with a week-to-week and night-to-night variability of sleep timing. The SSQ provides a fast assessment of the relevant sleep habits and moods before and during the pandemic. Still, in order to enable a clinical assessment of anxiety, anger, or depression, it remains appropriate to use standardized questionnaires such as the STAI, DASS (43), or STAXI (44), respectively. Even though the length of this instrument may complicate its interpretability, the use of various specific scales for every single mood construct assessed in the current study would be time consuming and difficult to perform in larger samples. Convenience sampling in the current study might have contributed to the under- or over-representation of some demographics. Specifically, the study enrolled a decreased proportion of elderly respondents, and presumably the respondents were mostly people familiar with the use of smartphones, tablets, or computers.

The SSQ enables fast screening that may be performed easily in various populations and provide complete data. As we recognized a shift in chronotype, while taking into account the associated poor sleep quality (45,46), more commonly reported sleep disorders (47) and the reported stability of these changes (5) in the population, interventions addressing these issues are warranted.

There is a growing awareness that deviated sleep habits and decreased sleep quality are linked with an increased risk for sleep disturbances. Even though variables such as mood, social media use, and altered sleep hygiene are often underscored as being relevant for development of sleep disorders, tools required to recognize and report such associations in the general adult population are scarce. Therefore, implementation of the SSQ to assess sleep habits, and especially circadian rhythm-related variables, during workdays and work-free days could improve the assessments of sleep problems and aid in the identification of people at risk for sleep disturbances. This may be of special importance at times of crisis.

Numerous questionnaires are currently available to evaluate sleep habits and sleep-related problems, but they lack comprehensiveness, which is the added value of SSQ. This instrument may be used in screening of sleep habits, while clearly differentiating the workdays and work-free days, which is lacking even in the most widely used standard and validated questionnaires. The SSQ allows an assessment of positive and negative moods, lacking a standardized assessment of anxiety, anger or depression, which requires the use of appropriate standardized questionnaires. Additionally, the SSQ questionnaire may be suited to evaluate the impact of the COVID-19 pandemic on sleep and mood in the general population.
