# Supplementary material for: The construction of the Split Sleep Questionnaire on sleep habits during the COVID-19 pandemic in the general population
Source: Croat Med J. 2022 Jun;63(3):299–309. doi: 10.3325/cmj.2022.63.299 (PMC9284018; doi:10.3325/cmj.2022.63.299)
Supplement: Supplementary Material [file CroatMedJ_63_s001.pdf]

For the future use of the SSQ, if there is a need for the online version of the questionnaire, contact the first author at linda.lusic@gmail.com in order to access a link you may use as an online form.

You are free to use the SSQ while citing the paper in which it was published (Lusic Kalcina et al., Croatian Medical Journal, 2022).

SLEEP HABITS AND MOOD SECTION

OF THE SPLIT SLEEP QUESTIONNAIRE (SSQ)

Dear respondents,
This questionnaire is completely anonymous and serves exclusively for scientific research which is planned and conducted by a group of scientists at the University of Split School of Medicine and the University of Mostar School of Medicine (adjust according to the institution using the questionnaire).

Please, fill this form sincerely in accordance with the questions that follow so the collected data could be properly analyzed and give a reliable and a credible illustration of the relevant information.

SLEEP HABITS

What time did you usually go to bed during weekday BEFORE the pandemic ? *

Example: 8.30 a.m.

What time did you usually wake up during weekday BEFORE the pandemic? *

Example: 8.30 a.m.

What time did you usually go to bed during weekend BEFORE the pandemic ? *

Example: 8.30 a.m.

What time did you usually wake up during weekend BEFORE the pandemic? *

Example: 8.30 a.m.

What time do you usually go to bed during weekday DURING the pandemic? *

Example: 8.30 a.m.

What time did you usually wake up during weekday DURING the pandemic? *

Example: 8.30 a.m.

What time do you usually go to bed during weekend DURING the pandemic? *

Example: 8.30 a.m.

What time did you usually wake up during weekend DURING the pandemic? *

Example: 8.30 a.m.

How long (in minutes) did it usually take for you to fall asleep BEFORE the pandemic: *

How long (in minutes) does it usually take for you to fall asleep DURING the pandemic: *

How did you usually wake up BEFORE the pandemic: *

Mark only one oval.

- Using an alarm clock
- By myself

How do you usually wake up DURING the pandemic: *

Mark only one oval.

- Using an alarm clock
- By myself

Did you usually take naps BEFORE the pandemic? *

Mark only one oval.

- Yes
- No

Do you usually take naps DURING the pandemic? *

Mark only one oval.

- Yes
- No

Did you wake up frequently at night BEFORE the pandemic? *

Mark only one oval.

- Yes
- No

If yes, how often have you experienced awakenings during night BEFORE the pandemic:

Mark only one oval.

- Rarely
- Occasionally
- Frequently
- Almost always

Do you wake up frequently at night DURING the pandemic? *

Mark only one oval.

- Yes
- No

If yes, how often do you experience awakenings during night DURING the pandemic:

Mark only one oval.

- Rarely
- Occasionally
- Frequently
- Almost always

Did you have insomnia BEFORE the pandemic? *

Mark only one oval.

- Yes
- No

Do you have insomnia DURING the pandemic? *

Mark only one oval.

- Yes
- No

Have you experienced difficulty falling asleep when going to bed in the evening BEFORE the pandemic? *

Mark only one oval.

- Yes
- No

Have you experienced difficulty falling asleep when going to bed in the evening DURING the pandemic? *

Mark only one oval.

- Yes
- No

Did you find yourself chronically tired before the pandemic? *

Mark only one oval.

- Yes
- No

Do you find yourself chronically tired during the pandemic? *

Mark only one oval.

- Yes
- No

Do you have any chronic disease? *

Mark only one oval.

- [ ] Yes
- [ ] No

If YES, which one?

____

MOOD SELF-ASSESSMENT

BEFORE the pandemic, how often did you feel: *

Mark only one oval per row.

	Not at all	Somewhat	Moderately	Very much so
Calm?	◯	◯	◯	◯
Rested?	◯	◯	◯	◯
Content?	◯	◯	◯	◯
Anxious (tense, preoccupied)?	◯	◯	◯	◯
Angry (irritatable)?	◯	◯	◯	◯
Scared?	◯	◯	◯	◯
Discouraged about the future?	◯	◯	◯	◯
Sad?	◯	◯	◯	◯

DURING the pandemic, how often do you feel: *

	Not at all	Somewhat	Moderately	Very much so
Calm?	◯	◯	◯	◯
Rested?	◯	◯	◯	◯
Content?	◯	◯	◯	◯
Anxious (tense, preoccupied)?	◯	◯	◯	◯
Angry (irritatable)?	◯	◯	◯	◯
Scared?	◯	◯	◯	◯
Discouraged about the future?	◯	◯	◯	◯
Sad?	◯	◯	◯	◯
