# Supplementary material for: The construction of the Split Sleep Questionnaire on sleep habits during the COVID-19 pandemic in the general population
Source: Croat Med J. 2022 Jun;63(3):299–309. doi: 10.3325/cmj.2022.63.299 (PMC9284018; doi:10.3325/cmj.2022.63.299)
Supplement: Supplementary Table 1 [file CroatMedJ_63_s002.pdf]

Supplementary Table 1. Cronbach's Alpha value of Sleep Habits section on sleep timing of the Split Sleep Questionnaire.

	Scale mean if item deleted	Scale variance if item deleted	Corrected item-total correlation	Cronbach's alpha if item deleted
Weekday bedtime before the COVID-19 pandemic	100.7	88.2	0.616	0.794
Weekday bedtime during the COVID-19 pandemic	100.4	80.1	0.578	0.793
Weekend bedtime before the COVID-19 pandemic	100.0	80.6	0.626	0.786
Weekend bedtime during the COVID-19 pandemic	100.2	74.3	0.462	0.830
Weekday waketime before the COVID-19 pandemic	116.4	95.3	0.301	0.826
Weekday waketime during the COVID-19 pandemic	116.0	88.4	0.539	0.800
Weekend waketime before the COVID-19 pandemic	115.3	82.9	0.695	0.780
Weekend waketime during the COVID-19 pandemic	115.1	80.4	0.687	0.778
