# Supplementary material for: The construction of the Split Sleep Questionnaire on sleep habits during the COVID-19 pandemic in the general population
Source: Croat Med J. 2022 Jun;63(3):299–309. doi: 10.3325/cmj.2022.63.299 (PMC9284018; doi:10.3325/cmj.2022.63.299)
Supplement: Supplementary Table 2 [file CroatMedJ_63_s003.pdf]

Supplementary Table 2. Cronbach's Alpha value of the Mood section of the Split Sleep Questionnaire.

		Scale mean if item deleted	Scale variance if item deleted	Corrected item-total correlation	Cronbach's Alpha if item deleted
Before the COVID-19 pandemic	Calm	14.06	37.29	0.45	0.89
	Rested	13.90	36.48	0.45	0.89
	Satisfied	14.12	37.47	0.45	0.89
	Anxious	14.48	35.79	0.50	0.89
	Angry	14.63	36.13	0.50	0.89
	Afraid	14.92	35.58	0.56	0.88
	Discouraged	14.82	34.82	0.57	0.88
	Sad	14.66	35.79	0.56	0.88
During the COVID-19 pandemic	Calm	13.84	35.27	0.60	0.88
	Rested	13.85	35.56	0.53	0.88
	Satisfied	13.94	35.66	0.61	0.88
	Anxious	14.24	34.32	0.58	0.88
	Angry	14.47	34.54	0.61	0.88
	Afraid	14.62	34.77	0.54	0.88
	Discouraged	14.54	33.23	0.64	0.88
	Sad	14.50	34.79	0.61	0.88
